# Using a lighter to heat a cautery

**Published:** 2011-09

**Authors:** Brian Savage

**Affiliations:** Haydom and KCMC Hospitals, Tanzania.

## Dear Editor

Those of us who are extracapsular cataract surgeons have all experienced delays in cauterizing the eye due to difficulty in lighting a spirit lamp containing methylated spirit with too much water mixed in it. What should be a simple and short stage of the operation becomes tense and prolonged, cautery may be inadequate, and there is inefficient use of anaesthetic time.

We have found that using a cigarette lighter for heating is a viable alternative.

In Tanzania, the cigarette lighter illustrated is easily available and can be purchased cheaply from local stores, costing only TZS 500 (around US $0.30). The flame effectively heats the ball of the Wordsworth cautery, and soot accumulation can be avoided if the cautery is held in the blue rather than the yellow part of the flame.

So far, we have tested two models of cheap lighter. Not all are suitable, as the top of the lighter becomes hot and parts may melt during prolonged use. For this reason also we recommend that surgical gloves are not worn by the person holding the lighter. When using a lighter of the type illustrated, we have found this method to be safe, easy to operate, and effective both in our own theatre and on outreach.

**Figure:**
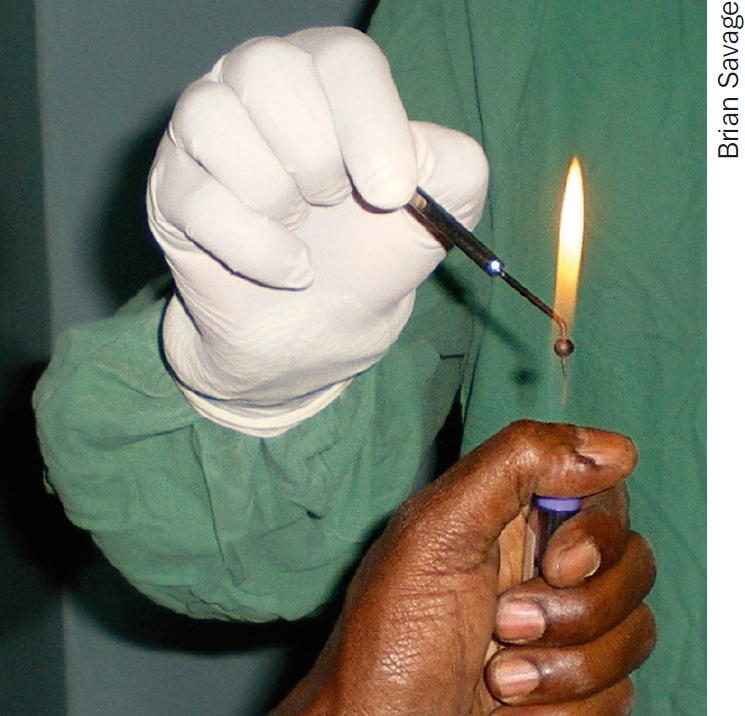
Wordsworth cautery being heated in blue part of the flame. (The flame here is turned much higher than normal for illustration purposes)

Using a lighter will also reduce the risks of fire in the theatre caused by gowns or drapes coming in contact with the flame of an unattended spirit lamp, when operator or assistant are absorbed by a challenging operation. I have seen this once: surgical gowns are surprisingly flammable!

In general anaesthetic situations, the usual precautions regarding inflammable gases and naked flames should be observed.

**Figure:**
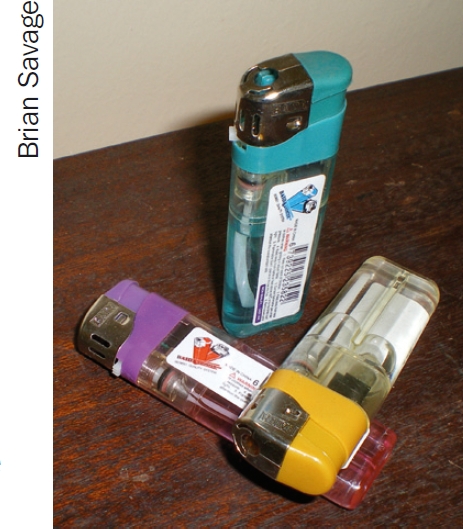
Examples of the lighter we have found effective

